# A collection of barcoded natural isolates of *Saccharomyces paradoxus* to study microbial evolutionary ecology

**DOI:** 10.1002/mbo3.773

**Published:** 2018-12-19

**Authors:** Clara Bleuven, Alexandre K. Dubé, Guillaume Q. Nguyen, Isabelle Gagnon‐Arsenault, Hélène Martin, Christian R. Landry

**Affiliations:** ^1^ Département de Biologie Université Laval Québec Québec Canada; ^2^ Institut de Biologie Intégrative et des Systèmes (IBIS) Université Laval Québec Québec Canada; ^3^ Big Data Research Center Université Laval Québec Québec Canada; ^4^ PROTEO, The Quebec Network for Research on Protein Function, Engineering, and Applications Québec Québec Canada; ^5^ Département de Biochimie de Microbiologie et de Bio‐informatique, Université Laval Québec Québec Canada; ^6^ Département des Sciences des aliments, Institut sur la nutrition et les aliments fonctionnels (INAF) Université Laval Québec Québec Canada

**Keywords:** barcoded yeast collection, competition assay, *Saccharomyces paradoxus*, wild yeast

## Abstract

While the use of barcoded collections of laboratory microorganisms and the development of barcode‐based cell tracking are rapidly developing in genetics and genomics research, tools to track natural populations are still lacking. The yeast *Saccharomyces paradoxus* is an emergent microbial model in ecology and evolution. More than five allopatric and sympatric lineages have been identified and hundreds of strains have been isolated for this species, allowing to assess the impact of natural diversity on complex traits. We constructed a collection of 550 barcoded and traceable strains of *S. paradoxus*, including all three North American lineages *SpB*, *SpC*, and SpC*. These strains are diploid, many have their genome fully sequenced and are barcoded with a unique 20 bp sequence that allows their identification and quantification. This yeast collection is functional for competitive experiments in pools as the barcodes allow to measure each lineage's and individual strains’ fitness in common conditions. We used this tool to demonstrate that in the tested conditions, there are extensive genotype‐by‐environment interactions for fitness among *S. paradoxus* strains, which reveals complex evolutionary potential in variable environments. This barcoded collection provides a valuable resource for ecological genomics studies that will allow gaining a better understanding of *S. paradoxus* evolution and fitness‐related traits.

## 1 INTRODUCTION

Fungal microbes are powerful models in genetics and molecular biology and increasingly so in ecological and evolutionary research (Bleuven & Landry, 2016; Koskella & Vos, 2015; Landry, Townsend, Hartl, & Cavalieri, 2006; Marsit et al., 2017). Among fungi, the genus *Saccharomyces*, particularly *Saccharomyces cerevisiae*, is well known for being used as a model system in the laboratory and for being closely associated with other human activities such as beer, wine, and bread making. However, the *Saccharomyces* genus also includes nonhuman‐associated species, found in natural habitats under limited human influence. Even if several studies have highlighted the evolutionary history of nondomesticated budding yeasts, their ecology and life history traits are still largely unknown (Boynton & Greig, 2014; Sampaio & Gonçalves, 2017).

**Figure 1:**
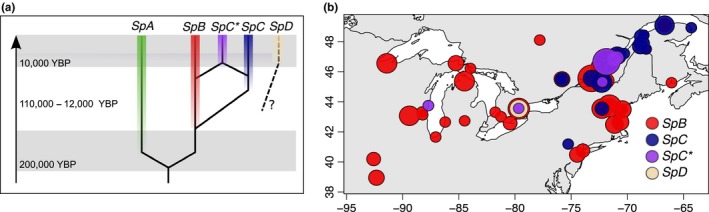
The *S. paradoxus *population structure and geographical distribution in North America. (a) Representation of the evolutionary history of the *S. paradoxus *North American lineages (Leducq et al., 2016). The European *SpA* and American lineages diverged about 200,000 years ago. It is hypothesized that *SpB* and *SpC* were in allopatry during the last glaciation from 110,000 to 12,000 before present (BP). A secondary contact between *SpB* and *SpC* would have occurred after the glacial retreats, leading to the formation of SpC* by hybridization. The *SpD* clade was identified recently, and its origin is not yet elucidated (Xia et al., 2017). (b) Geographical distribution of the *S. paradoxus* strains used in this study. Circle size is proportional to the number of strains at the location

*Saccharomyces cerevisiae*'s closest relative, *S. paradoxus*, is an emerging model to study fungal natural history, ecology, and evolution (Hénault et al., 2017; Replansky, Koufopanou, Greig, & Bell, 2008). *S. paradoxus* is one of the nondomesticated species of the genus *Saccharomyces*. It has been mainly isolated from deciduous trees and associated soils (Charron, Leducq, Bertin, Dube, & Landry, 2014a; Naumov, Naumova, & Sniegowski, 1998). Genetic diversity within this species is structured into five main lineages in North America: *SpA*, originally from Europe but recently introduced in North America, and *SpB*, *SpC*, SpC*, and *SpD* that are endemic to North America (Figure 1a). Other lineages have been identified worldwide, from Far East Asia to Hawaii (Hénault et al., 2017; Kuehne, Murphy, Francis, & Sniegowski, 2007; Leducq et al., 2016; Liti et al., 2009; Xia et al., 2017). The most recent research focuses on the endemic North American lineages *SpB*, *SpC*, and SpC* as models for speciation and hybridization (Charron, Leducq, & Landry, 2014b; Leducqet al., 2017, 2016) and for adaptation to climatic conditions (Eberlein et al., 2017; Leducq et al., 2014). The population structure observed in North America shows that these lineages show partial postzygotic reproductive isolation. Also, no evidence of first‐generation hybrids has been found in nature so far, suggesting that *SpA*, *SpB*, and *SpC* may actually represent fully isolated species (Charron, Leducq, & Landry, 2014b). Finally, Xia et al. (2017) recently described an additional highly diverse group, designated as the *SpD* lineage.

These lineages occupy a large geographic region with extensive environmental variation. It has been shown that *SpB*, *SpC*, and SpC* display a distribution closely linked to their different ranges of temperature tolerance, therefore potentially reflecting ecological specialization (Leducq et al., 2014, 2016; Figure 1b). The lineages perform differently at high temperature and do not survive equally to freeze–thaw cycles, with southern populations outperforming northern ones. They also appear to diverge in terms of performance when grown on limiting nutrient media with different carbon or nitrogen sources (Leducq et al., 2016; Samani et al., 2015). The molecular basis of this ecological specialization has been examined and candidate genes have been identified as potential key players (Eberlein et al., 2017). For example *GRS2*, which codes for an aminoacyl tRNA‐synthetase, is expressed at high temperature and its protein level differs in abundance between the *SpB* and *SpC *lineages. Allele swapping experiments revealed that protein‐coding changes at this gene could be partly responsible for the inability of *SpC* to grow at high temperature.

Most studies have compared the fitness of different strains in controlled conditions using colonies grown isolated from each other on solid media. Being able to compare their fitness when in contact with each other would eventually allow to measure direct interactions among lineages or strains or interactions with other microbial species in a shared environment. One approach that has been developed recently for the study of model organisms is the use of DNA barcodes to track strains individually within a pool (Mazurkiewicz, Tang, Boone, & Holden, 2006). For more than a decade, barcoded yeast collections have been a powerful genomic tool to advance our knowledge of genomics and cell biology (Giaever & Nislow, 2014). The method relies on a unique short DNA segment inserted in a strain, which enables it specific identification. Using barcode sequencing (also known as Bar‐Seq; Filteau, Charron, & Landry, 2017; Gresham et al., 2011; Robinson, Chen, Storey, & Gresham, 2014; Smith et al., 2009), relative fitness is measured within and between conditions by monitoring the relative abundance of each barcode through time in a mixed pool of strains. The various applications of the *S. cerevisiae* knockout collection (Giaever et al., 2002), in which one gene is deleted and replaced by an antibiotic resistance cassette flanked by two unique barcodes, is a relevant example of the diversity of uses this tool can offer (Nislow et al., 2015; Novo et al., 2013; Sliva, Kuang, Meluh, & Boeke, 2016; VanderSluis et al., 2014). Other yeasts have been barcoded with similar approaches, for instance the collection of *Schizosaccharomyces pombe* insertion mutants (Chen, Hale, Ciolek, & Runge, 2012) and various isolates of *S. cerevisiae* (Cubillos, Louis, & Liti, 2009; Maclean et al., 2017).

Here, we barcoded a collection of North America wild *S. paradoxus* strains to facilitate the study of natural diversity in controlled conditions. This collection includes 198 *SpB*, 64 *SpC*, 47 SpC*, and 5 *SpD *barcoded strains. To illustrate the use of this collection, we performed an experiment in which the strains were pooled and competed in rich medium (YPD) at 25°C and 35°C and in synthetic medium supplemented with proline at 25°C. This allowed to measure each lineage's and each strain's relative fitness. We demonstrate that the different lineages, and strains within lineages, show extensive variation in fitness in these conditions, including genotype‐by‐environment interactions.

## 2 MATERIAL AND METHODS

### 2.1 Barcode and resistance cassette amplification

**Figure 2:**
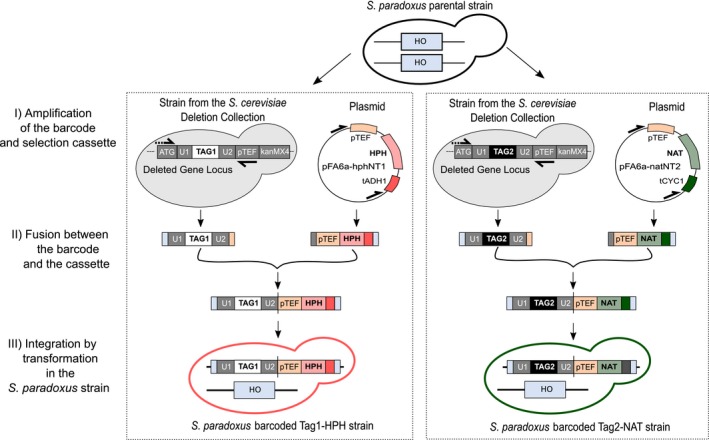
Integration protocol to barcode natural *S. paradoxus* strains. Two different barcodes (Tag1 and Tag2) were assigned to each individual strain according to its associated antibiotic resistance cassette, hygromycin B (HPH), or nourseothricin (NAT). The integration method involves three steps: (I) Barcode amplification from the *S. cerevisiae* deletion collection and antibiotic resistance cassette amplification from plasmids pFA6a‐hph‐NT1 for hygromycin B (HPH) and from pFA6a‐nat‐NT2 for nourseothricin (NAT); (II) Barcode fusion by PCR with the antibiotic resistance cassettes; (III) Barcode insertion in *S. paradoxus* by transformation and homologous recombination. Each strain was barcoded in two copies, one with the Tag1‐HPH module and the other with the Tag2‐NAT module, each time with a unique barcode (Tag)

Barcodes from the *S. cerevisiae* deletion collection (Giaever et al., 2002) were amplified and combined with the hygromycin B (HPH) or the nourseothricin (NAT) resistance cassette and inserted by transformation and homologous recombination at the HO locus of selected diploid *S. paradoxus *strains (Figure 2, step I). The HO locus was chosen as the barcode integration site because it is a common neutral replacement site in laboratory strains. Indeed, HO is not required for growth and the deletion has no detectable effect on vegetative growth when replaced with a resistance cassette (Baganz, Hayes, Marren, Gardner, & Oliver, 1997). The endonuclease encoded by HO is responsible for mating‐type switching and is consequently inactive in diploids (Haber, 2012). The deletion in diploids will allow the production of stable haploids by dissection of the barcoded strains if needed.

Genomic DNA was extracted following a protocol modified from Looke, Kristjuhan, and Kristjuhan (2011). Strains from the *S. cerevisiae* deletion collection were printed onto arrays of 384 colonies on solid yeast peptone dextrose (YPD) with 10 g/L of yeast extract, 20 g/L of tryptone, and 20 g/L of glucose, as outlined in Rochette et al. (2015) using a BMC‐BC robotic platform (S&P robotics, North York, Canada). Cells were picked from YPD plates and suspended in 50 μl of 200 mM LiOAc, 1% SDS solution in a 96‐well PCR plates before incubation at 70°C for 15 min in a MasterCycler ProS (Eppendorf, Hamburg, Germany). 75 μl of 95% ethanol was then added, and the samples were mixed before a first centrifugation at 926 *g* for 48 min (5810R, Eppendorf, Hamburg, Germany). Supernatants were removed, and cell pellets were washed with 75 μl of 70% ethanol. After a second centrifugation at 926 *g* for 48 min, cell pellets were dried and dissolved in 50 μl of sterile water. Cell debris were spun down by centrifugation at 926 *g* for 3 min and 2 μl of the supernatant was used for barcode amplification by PCR. All oligonucleotides used in this study are listed in Supporting Information Table S2. For the amplification of the barcodes, forward primers were designed to be homologous to the upstream region of the HO gene, taking into consideration the divergence between the *S. paradoxus* lineages (Leducq et al., 2014). Reverse primers were homologous to the shared region of the HPH and NAT resistance cassette module. The 15 μl PCR reaction mix contained 1 × PCR buffer, 0.3 mM of dNTPs, 0.3 μM of each primer, 0.18 U of Kapa Hifi DNA polymerase (Kapa Biosystems Inc., Wilmington, MA, USA), and 2 μl of genomic DNA. All PCR amplifications were performed in a MasterCycler ProS (Eppendorf, Hamburg, Germany). Cycling protocol details are listed in Supporting Information Table S4. In 141 cases, we were not able to amplify the barcode of the initially selected deletion strain from the *S. cerevisiae* collection, so a second one was used and reported in our database (Supporting Information Table S1). Two different and unique barcodes were assigned to each *S. paradoxus *strain and associated with the HPH or NAT resistance cassette to form the Tag1‐HPH and Tag2‐NAT copies.

Plasmids pFA‐hph‐NT1 and pFA6‐nat‐NT2 (Janke et al., 2004) were used to amplify the HPH and NAT resistance cassettes by PCR. Each 50 μl PCR mix contained 1 × PCR buffer, 0.3 mM of dNTPs, 0.3 μM of each primer, 0.6 U of Kapa Hifi DNA polymerase (Kapa Biosystems Inc., Wilmington, MA, USA), and 0.2 ng/μl of plasmid (pFA‐hph‐NT1 or pFA6‐nat‐NT2). Forward primers were designed to target the resistance cassette module, and reverse primers were designed to target the downstream region of the HO gene, again taking into consideration the sequence divergence present among the lineages.

### 2.2 Fusion PCR

The PCR products containing the barcodes were fused by PCR with the resistance cassettes, HPH or NAT (Figure 2, step II) to form the Tag1‐HPH and Tag2‐NAT modules. Each 15 μl PCR mix contained 1 × PCR buffer, 0.3 mM of dNTPs, 0.3 μM of each primer, 0.18 U of Kapa Hifi DNA polymerase (Kapa Biosystems Inc., Wilmington, USA), 5 μl of 1/10 barcode amplification PCR product, and 0.5 μl of 1/10 cassette amplification PCR product. Following this procedure, fusion PCRs were successfully performed for the cassettes to be used in the *SpB* and *SpD* lineages. However, due to a lower transformation efficiency following the fusion, new PCR primers had to be designed to have longer sequence homology for the *SpC *and SpC* lineages (Supporting Information Table S2). This lead to a higher rate of successful transformations.

### 2.3 Barcodes insertion

All natural strains used in this study are listed in Supporting Information Table S1. Their geographic distribution is represented in Figure 1. Competent cells and transformation were performed as in Gietz and Woods (2002) with the following modifications: cells were grown overnight in 5 ml of YPD at 30°C without agitation, diluted to an OD_595_ of 0.15/ml, and grown again to an OD_595_ of 0.4–0.7/ml. Each culture was harvested by centrifugation at 500 *g* for 5 min, and the pellets were successively washed with 1 ml of sterile water and 1 ml of sterile SORB (100 mM LiOAc, 10 mM Tris pH8.0, 1 mM EDTA, and 1 M Sorbitol). Cells were resuspended in 18 μl of SORB and 2 μl of salmon sperm carrier DNA (10 mg/ml). Samples of competent cells were then split in two and stored at −80°C. For transformation, 8 μl of Fusion PCR and 20 μl of thawed competent cells were mixed in 96‐well PCR plates. Plate mixture was added (100 μl of 100 mM LiOAc, 10 mM Tris‐HCl pH 8, 1 mM EDTA/NaOH pH8, 40% PEG3350), and cells were incubated at room temperature for 30 min. 7.5 μl of DMSO was added, and cells were incubated for 30 min at different temperatures depending on their lineage: at 37°C for *SpC* and SpC* strains and 42°C for *SpB* and *SpD* strains. After centrifugation and removal of the supernatant, 100 μl of YPD was added before a last incubation of at least 5 hr at 30°C. Cells were then plated on YPD media supplemented with 250 μg/ml of hygromycin B or 100 μg/ml of nourseothricin and incubated at 30°C for 3 days. The growing colonies were transferred on solid YPD media supplemented with 250 μg/ml of hygromycin B or 100 μg/ml of nourseothricin and grown for 1 day at 30°C.

Confirmation of cassette insertion was done by colony PCR. Colonies grown after the last step were then resuspended in 40 μl of 20 mM NaOH and incubated 20 min at 95°C. Samples were centrifuged 5 min at 1,556 *g* (5430, Eppendorf, Hamburg, Germany), and 2 μl of the supernatant was added to a PCR mix. Each 20 μl of the PCR mix contained 1 × PCR buffer, 0.2 mM of dNTPs, 0.2 μM of primer F and 0.1 μM of both primers R, CLO5–28 and O1–37 or CLO5–28 and O1–38, 1.5 mM of MgCl_2_ and 0.6 U of DNA polymerase (Bioshop, Burlington, Canada). When validated, barcoded strains were stored at −80°C in YPD medium supplemented with 250 μg/ml of hygromycin B or 100 μg/ml of nourseothricin and 25% glycerol. As several strains had abnormal colony morphology during the following experiments, which we hypothesized could be bacterial contamination, all barcoded strains were plated on YPD media supplemented with 12.5 μg/ml of chloramphenicol and incubated at 30°C for 2 days and then stored at −80°C in YPD medium supplemented with 250 μg/ml of hygromycin B or 100 μg/ml of nourseothricin and 25% glycerol.

Following colony PCR, Sanger sequencing of 539 out of the 594 transformed barcodes was used to verify that the expected barcodes were inserted at the HO locus (Plateforme de séquençage et génotypage des génomes, CHUL, Quebec, Canada; see Supporting Information Table S1).

### 2.4 Barcoded strains phenotypic analysis

The 370 parental strains and 594 barcoded strains of the collection were assembled in two arrays (omnitrays, 86 mm × 128 mm Petri dish) on solid YPD medium (Rochette et al., 2015). One contained all the *SpB* and *SpD* strains while *SpC* and SpC* strains were on the other second array. These arrays were then replicated on eight YPD plates each, which were incubated at 25°C and at 35°C for two days. As in Eberlein et al. (2017), colonies were then transferred by replication on new plates and incubated for three days at 25°C and 35°C. Plate pictures were taken with an EOS Rebel T3i camera (Canon, Tokyo, Japan) at different intervals right after the replication (hereafter referred to as *t*_0_) and after 3, 6, 9, 13, 19 hr (hereafter referred to as *t*_5_), 23, 26, 29, 46, and 53 hr of incubation. Colony size was measured using ImageJ 1.45s (National Institutes of Health, Bethesda, USA; https://imagej.nih.gov/ij/) by counting integrated pixel intensities of colonies as described in Diss, Dubé, Boutin, Gagnon‐Arsenault, and Landry (2013). Colony sizes after 19 hr were used for further analysis to capture growth rates before saturation. Growth of strain at *t*_5_ was estimated by difference between the log_2_ transformed size of the colonies at *t*_5_ and *t*_0_. Following Sanger sequencing of the barcodes, we removed from the analysis strains sharing the same barcodes (*n* = 44) as well as strain LL12_003 because it had abnormal colony morphology. Among the 549 remaining strains, only the ones barcoded with both the Tag1‐HPH and Tag2‐NAT were considered for further analysis, that is, 151 *SpB*, 52 *SpC*, 32 *SpC*, and 2 *SpD* strains. We performed Kruskal–Wallis tests to test whether the barcodes affected growth in the different conditions. All statistical analyses and data handling were performed using RStudio version 1.0.153 from R Core Team (2017).

### 2.5 Barcoded strain competition assay

The *S. paradoxus* barcoded strains were pooled as in Smith et al. (2011) with the following modifications: the collection was replicated from glycerol stock onto YPD plates in a 384‐array format and incubated for 2 days at 30°C. To maximize their growth before pooling, the strains were transferred for a second round of incubation on YPD plates for 2 days at 30°C. The set of *SpB* and *SpD* barcoded strains were first pooled together in an intermediate pool and *SpC* and SpC* in another one. The two pools were combined into a single one to obtain a mix of the four lineages. Cells were adjusted to concentrations equivalent to 50 optical density (OD_595_) in YPD + 25% glycerol, and the pool was aliquoted in several 1 ml tubes and stored at −80°C. Strains LL12_028 (Tag1‐HPH and Tag2‐NAT copies, *SpB*), LL12_003 (Tag1‐HPH and Tag2‐NAT copies, *SpB*), LL13–025 (Tag1‐HPH and Tag2‐NAT copies, *SpB*), LL11_002 (Tag1‐HPH copy, *SpC*), LL12_004 (Tag1‐HPH copy, *SpC*), and LL12_007 (Tag1‐HPH copy, *SpC*) were removed because they had abnormal colony morphologies suggesting that they were either contaminated or were affected by the transformation.

**Figure 3:**
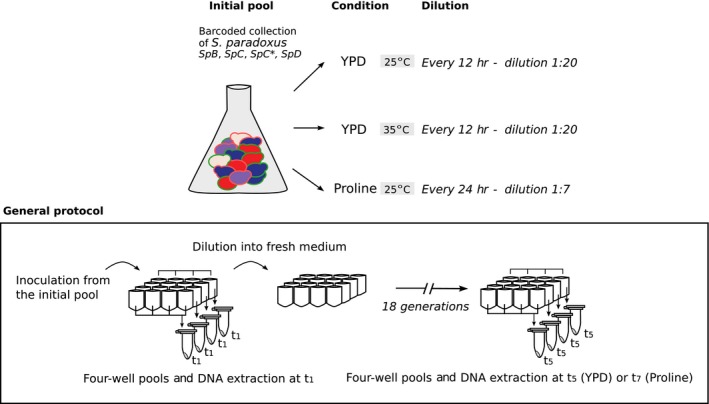
Competition assay using the barcoded strains. The initial pool contains all barcoded strains with either the Tag1‐HPH (red border) or the Tag2‐NAT (green border) module from the *SpB* (red), *SpC* (blue), SpC* (purple), and *SpD* (beige) lineages. Wells of a deep‐well plate were inoculated with the pool in three conditions: YPD at 25°C, YPD at 35°C, and proline medium at 25°C. Until the cultures reach 18 generations, each well was diluted to keep cells in exponential growth. At the end of the experiment, wells were pooled by group of four to extract DNA. Samples were collected at *t*_1_ and points *t*_5_ for the YPD medium and *t*_7_ for the proline medium

Competition assays were carried out in 96‐deep‐well plates in three conditions: YPD at 25°C and 35°C, and synthetic minimal medium supplemented with proline (1.74 g/L of Yeast Nitrogen Base without ammonium sulfate, 20 g/L of glucose, 5 g/L of l‐proline) at 25°C without agitation (Figure 3). The experiment was initiated by the inoculation of 32 wells with the pool at OD_595_ = 0.08. Dilutions to fresh YPD at 25°C and 35°C were done every 12 hr by transferring 50 μl into new wells filled with 950 μl of fresh YPD until the cultures reached 18 generations. Dilutions in the proline medium at 25°C were done every 24 hr by transferring 150 μl into new wells filled with 850 μl of fresh medium. Samples from initial to final time points were conserved by resuspension in sterile water after centrifugation and elimination of the supernatant and stored at −80°C. The OD was monitored using a plate reader (Infinite F200 Pro Tecan, Zürich, Switzerland) before each dilution. We estimated the number of generations (cell doubling) for each time point by the difference in log_2_ of cell counts before dilution and the initial cell count:

$$\text{Estimated number of generations at}\;t_{i}=\log_{2}\left({\text{cell number}}_{i}\right)-\log_{2}\left({\text{cells number}}_{i-1}\right)$$

with ${\text{cell number}}_{i}={\text{DO}}_{i}\times 10^{7},$ corresponding to the estimated cell number in the well at *t* *= i* and with ${\text{cell number}}_{i-1}={\text{DO}}_{i-1}\times 10^{7}\times \text{dilution rate,}$ corresponding to the estimated cell number inoculated after the dilution at *t *= *i* − 1 with dilution rates corresponding to 1/20 for YPD and 1/7 for proline medium. To estimate the total number of generations of the assay, we added the number of generations of each time point for each condition and stopped the assay when the total reached 18 generations. This number of generations was used to minimize the accumulation of spontaneous mutations while maximizing the resolution of the assays as in Filteau et al. (2017). The experiment conducted in YPD at 25°C and 35°C reached 18 generations at the time point *t*_5_ and the experiment in proline medium at *t*_7_. We refer to *t*_1_ as our initial time, equivalent to five generations in YPD and 3.5 generations in proline medium.

To limit stochastic effects, we pooled four different wells at the end of the experiment to obtain at least four final replicates for each condition at the initial and final time points (YPD at 25°C at *t*_1_ and *t*_5_, YPD at 35°C at *t*_1_ and *t*_5_, and proline medium at 25°C at *t*_1_ and *t*_7_). We extracted DNA from these pools using a protocol adapted from Amberg, Burke, and Strathern (2005) with the following modifications: after centrifugation, the supernatants were removed and the cells were resuspended in 200 μl of lysis buffer (2% triton 100X, 1% SDS, 100 mM NaCl, 10 mM Tris‐Cl (pH 8.0), 1 mM Na_2_EDTA) and 200 μl of a mix of 50% phenol, 48% chloroform, and 2% isoamyl alcohol and then 100 μl of glass beads (425–600 μm) were added. The cultures were vortexed for 4 min using a Thermomix (Scientific Industries, Inc., Bohemia, USA) and centrifuged at 16,100 *g* for 5 min (5415R, Eppendorf, Hamburg, Germany). The aqueous layer was transferred to a new tube, and an additional phenol–chloroform extraction was performed. After the centrifugation step, the aqueous layer was transferred to a new tube and 1/10 volume of 3 M sodium acetate pH 5.2 and two volumes of 95% ethanol (EtOH) were added. Samples were incubated at −20°C for 15 min and centrifuged for 15 min at 4°C. The supernatants were removed, and the cell pellets were resuspended in 400 μl of TE buffer. 3 μl of a 10 mg/ml RNase A solution (Bio Basic, Inc., Markham, Canada) was added, and the samples were incubated at 37°C for 5 min. After incubation, 40 μl of 3 M pH 5.2 sodium acetate and 800 μl of 95% EtOH were added. The tubes were incubated at −20°C for 15 min and centrifuged for 15 min at 16,100 *g* at 4°C. The supernatant was removed, the cell pellets were dried by evaporation at 37°C, and DNA samples were resuspended in 50 μl water. DNA concentration was measured using a Nanodrop 2000c (Thermo‐Fisher Scientific, Waltham, USA) and adjusted to 10 ng/μl for further use.

### 2.6 Barcode sequencing

Forward and reverse primers were used for multiplex sequencing with Ion Torrent technology using predefined indexes (Faircloth & Glenn, 2012) and newly designed ones (listed in Supporting Information Table S2). Each 25 μl PCR reaction mix sample contained 1 × PCR buffer, 0.3 nM of dNTP, 0.5U of Kapa HiFi DNA polymerase (KAPA Biosystems Inc., Wilmington, MA, USA), 50 ng of genomic DNA, and 0.2 μM of each primer. PCR products were pooled and purified using the KAPA Hyper Prep Kit from Illumina (KAPA Biosystems, Wilmington, USA), and quality was assessed and sequenced at the IBIS sequencing platform on an Ion Proton instrument (Thermo‐Fisher Scientific, Waltham, USA) according to the manufacturer's instructions.

### 2.7 Barcode sequence analysis and quantification

All possible expected PCR products including the dual‐index and the barcodes were concatenated with NNNNN spacer. This reference sequence was used for mapping barcoded sequences using Geneious version R6 (Kearse et al., 2012). After quality check filtration, sequence fragments were selected by length, excluding sequences below 90 bp. From the 48,097,196 resulting barcode sequences, 40,634,976 were successfully mapped on the reference sequence with the following parameters: word length 20, index word length 15, repeated words ignored 50, maximum mismatches per reads 6%, allowed gap 4%, maximum gap size 2, and maximum ambiguity 4. Reads with multiple best matches were excluded. The percentage of excluded sequences obtained was 15.51%. All libraries had more than 140,000 reads. Following Sanger sequencing of the barcodes, we removed from the analysis strains sharing the same barcodes (*n* = 44) as well as the strains for which the barcode failed to be sequenced (*n* = 18). Among the remaining strains, only strains barcoded with both Tag1‐HPH and Tag2‐NAT and that had more than 100 reads at *t*_1_ in each replicate were considered for further analysis (See Supporting Information Table S1 for strains considered).

Relative fitness (ω) was calculated with the following equation (Filteau et al., 2017; Qian, Ma, Xiao, Wang, & Zhang, 2012):

$$\omega ={\left(\frac{P_{\text{final}}}{P_{\text{initial}}}\right)}^{\frac{1}{\text{generations}}}$$

where *P*_final_ is the frequency of the strain at the final time (*t*_5_ or *t*_7_) and *P*_initial_ is the frequency of the strain at the initial time (*t*_1_) of the competition assay. The frequency for each strain was calculated as its number of reads divided by the total number of reads in the library considered. We used 18 generations for the calculations. For each strain, the relative fitness value of its copies, Tag1‐HPH and Tag2‐NAT, was estimated using the median relative fitness of the four replicates. The global relative fitness of each strain within a given condition was calculated by using the mean value of the Tag1‐HPH strain and Tag2‐NAT relative fitness. Finally, to determine the fitness of each lineage in each condition, the average fitness of all strains belonging to each lineage was calculated. Twelve outlier strains were removed from the analysis because of their highly differential fitness between their two barcoded copies, Tag1‐HPH and Tag2‐NAT. To define the filtering threshold, the mean and the standard deviation (*SD*) of the difference between the fitness of the Tag1‐HPH and Tag2‐NAT copies of all the strains were calculated. Strains with a difference value higher or lower than mean ± (2.5 × *SD*) were removed.

## 3 RESULTS

### 3.1 Transformation and integration of the barcodes in the *S. paradoxus* strains

The *S. cerevisiae* deletion collection was used to amplify unique barcodes before integration in the *S. paradoxus* strains. This collection consists of strains in which nonessential genes have been individually replaced with a *KanMX* module, which confers resistance to geneticin, and two flanking unique DNA barcodes of 20 bp labeled as uptags and downtags (Giaever et al., 2002). Because the uptag barcodes were previously sequenced in Filteau et al. (2017) and presented fewer discrepancies with the database of the deletion collection than the downtag, we used the uptags only. Changes in barcode sequences that were detected by Filteau et al. (2017) are listed in Supporting Information Table S1.

From one to three rounds of transformation were performed to insure barcode insertion in a maximum of strains. This was achieved successfully for more than half of the parental strains (Supporting Information Table S3). Integration failure could be due to polymorphisms in the flanking regions of HO locus where homologous recombination takes place or at the loci for PCR confirmation or to variation among strains in their level of competence for transformation. Proper integration could not be confirmed for every strain, suggesting that the barcode and the selection cassette were not always at the appropriate genome location. Such strains were not further considered.

Thus, the *Saccharomyces paradoxus* barcoded collection consists of 550 strains from the *SpB*, *SpC*, SpC* lineages, and the *SpD* group, either barcoded in two distinct copies, with the Tag1‐HPH and Tag2‐NAT module (*n* = 238) or in single copy with a Tag1‐HPH (*n* = 34) or the Tag2‐NAT module (*n* = 40; see details in Table 1). The parental *SpD* strains were in small number, and two out of nine *SpD* strains were obtained in one copy, and three out of nine *SpD* strains were barcoded with both copies. The sequencing of 539 inserted barcodes showed that seven barcodes carried a mutation, 18 barcoded were not likely legible due to sequencing errors and 49 barcodes were not expected to be in the constructed strains. These could be errors of barcodes in the *S. cerevisiae* collection or contamination that took place during the experiment. Within these different barcodes, 19 were usable as they were not already associated to a *S. paradoxus* strain. However, we had to eliminate from the collection 44 strains that had a common barcode. Among the 594 transformed strains, 550 remained in the collection (93%).

**Table 1:** Number of *Saccharomyces paradoxus* barcoded strains in the collection

Lineage	Initial parental strains	Tag1‐HPH barcoded strains	Tag2‐NAT barcoded strains	Both barcodes
*SpB*	247	167	183	152
*SpC*	64	58	57	53
SpC*	50	42	36	31
*SpD*	9	5	2	2

### 3.2 Comparison of the growth between the barcode and parental strains

**Figure 4:**
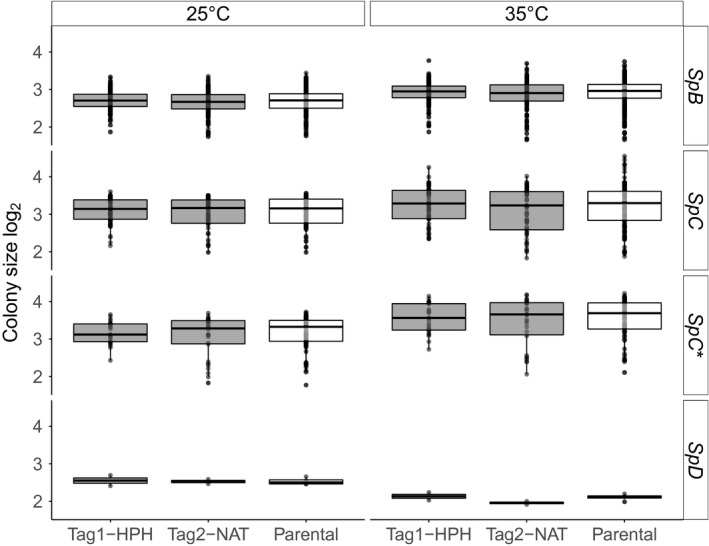
Barcoded strains show growth similar to that of parental strains on solid medium. Growth is inferred from colony size measurements after a 19‐hr incubation period on solid YPD medium at 25°C and 35°C. Eight replicates were performed for each strain. No significant differences were observed between the hygromycin B (HPH) barcode, the nourseothricin (NAT) barcode, and the WT strains at both temperatures for all lineages (Kruskal–Wallis tests, *p*‐values > 0.2)

To confirm that barcode insertion had no significant effect on growth compared to parental strains, a growth experiment was performed on solid rich medium (YPD) at 25°C and 35°C. Conditions were selected according to previous studies in which growth differences between lineages were observed (Leducq et al., 2016). For all lineages, no significant differences between the barcode and their parental strains were found (Kruskal–Wallis tests, Figure 4 and Supporting Information Table S5).

### 3.3 Competition assay

To test if quantitative barcode sequencing could be used with our collection and if it is adequately sensitive to characterize mean relative fitness of the lineages as well as individual relative fitness, we performed a competition assay in three specific conditions: YPD at 25°C, 35°C and proline medium at 25°C. These conditions were shown to differentiate the three lineages in previous studies (Charron & Landry, 2017; Leducq et al., 2016).

For each condition, read counts were highly reproducible across replicates (Supporting Information Figure S1, Pearson's *r* = 0.92–0.99, *p* < 0.01). After sequence data filtering, 206 out of the 238 strains barcoded in two copies were detected (for details, see Supporting Information Table S6) and 193 were used in the analysis, after eliminating the strains with highly differential fitness between their two tag modules. This suggests that a slightly higher sequencing depth would be required to cover the entire collection in future experiments.

**Figure 5:**
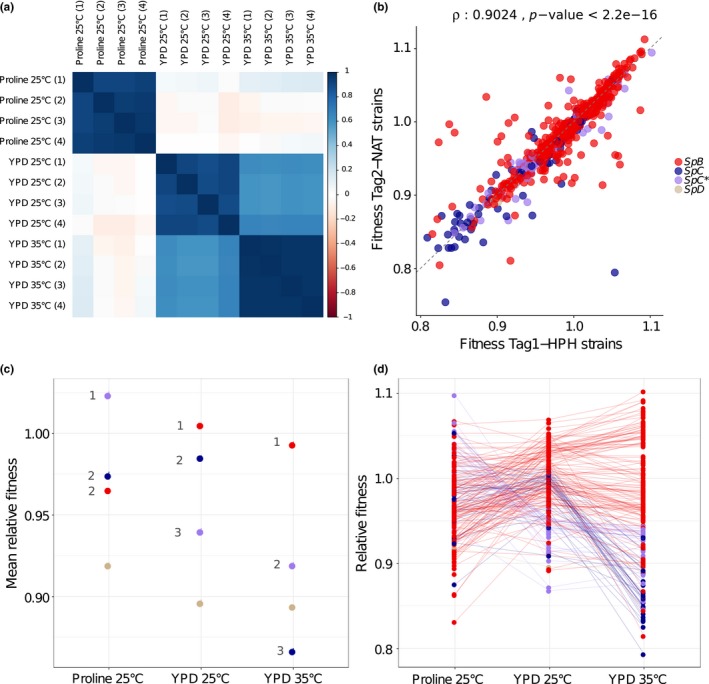
Relative fitness of strains and lineages of *S. paradoxus* assessed by barcode sequencing. (a) Correlation of relative fitness values between replicates and conditions. (b) The fitness estimates across experiments strongly correlate between the two modules Tag1‐HPH and Tag2‐NAT (Spearman's *ρ*: 0.9024, *p*‐value < 2.2e^−16^). (c) Average fitness of lineages in each condition. A Kruskal–Wallis test followed by a Dunn posthoc test was performed to compare the fitness of the *SpB*, *SpC*, and SpC* lineages. Numbers represent the fitness classes within each experimental condition among the lineages (see Supporting Information Table S7 for details). (d) Individual fitness values in each condition for the different lineages. Although absolute values cannot be compared between conditions, the relative values can be compared within conditions. The lines connect the same strains in two conditions. The average value of the estimates for the two tag modules is shown

The correlation of individual fitness values was examined to verify consistency between replicates and conditions (Figure 5a). A strong correlation (above 0.8) between the replicates within conditions was observed, and a much weaker correlation (less than 0.18) was observed between conditions, in particular between the YPD and proline media. The correlation coefficients between different incubation temperatures in YPD are intermediate, from 0.60 to 0.68, suggesting that a change in temperature from 25°C to 35°C has a weaker effect on the relative growth rates than a change in nutrient sources. In most cases, the fitness values estimated from the Tag1‐HPH and Tag2‐NAT copies strongly correlate with each other (Figure 5b; Spearman's ρ: 0.90, *p*‐value < 2.2 e^−16^).

Because of this strong correlation, we used the mean of the two values to estimate the global relative fitness of each strain in each condition. The analysis (Figure 5c) shows that the lineages significantly differ in average fitness within conditions and that the ranking changes between conditions. Whereas SpC* outperforms the other lineages in proline medium (Dunn posthoc tests, *p*‐values < 0.01, see details in Supporting Information Table S7), *SpB* occupies this position in the two other conditions. The fitness ranking also changes dramatically between 25°C and 35°C in YPD. Whereas *SpC* outperforms SpC* at 25°C (Dunn posthoc test, *p*‐value < 0.01), the ranking is reversed at 35°C (Dunn posthoc test, *p*‐value < 0.01). These results are consistent with previous observations showing that the hybrid species SpC* outperforms both parental species when using proline as a major nitrogen source and outperforms the parental species *SpC *at high temperature in rich media (Leducq et al., 2016). The two lineages *SpC *and the hybrid SpC* therefore show relative fitness values that are highly variable across condition. Although only two representative barcoded strains were obtained for *SpD*, this group appears to perform poorly compared to the other lineages. Altogether, these results indicate that genetic variation in performance is highly condition‐dependent, revealing genotype‐by‐environment interactions at the level of the lineages.

The use of individual barcodes also allows examining individual fitness values (Figure 5d). The analysis shows the magnitude of the intra‐lineage variation in each condition, which is particularly extreme in the *SpB* lineage. Although on average *SpB *strains outperform other strains at high temperature for instance (Figure 5c), individual *SpB* fitness values cover almost the entire range of values observed for the other lineages, suggesting that there is a large variation within *SpB *in fitness at high temperature. An extensive change in ranking among individual strains was also observed across conditions, supporting again the presence of complex genotype‐by‐environment interactions but at the individual strain level.

**Figure 6:**
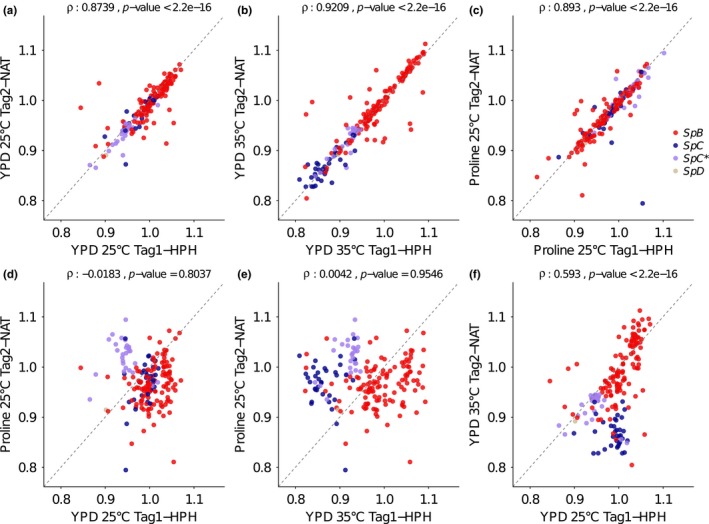
Genotype‐by‐environment interaction for fitness. The interaction between genotype and environment was investigated by analyzing the correlation between the strain fitness within (a, b, and c) and between (d, e, and f) conditions, considering the two tag modules as biological replicates. Fitness correlation is systematically high between tag modules within condition comparisons. This shows the extent of noise caused by strain transformations and/or biases or noise in barcode quantification (a, b, and c) and the maximum correlation possible between conditions. Correlations between conditions are systematically lower, showing a major effect of growth conditions in relative fitness among strains

To show that this variation is more important than the variation introduced by the transformation of the two barcodes and the overall experimental noise, we compared the extent of the correlation between the two sets of modules Tag1‐HPH and Tag2‐NAT within conditions (Figure 6a–c) and between conditions (Figure 6d–f). Comparisons within condition reveal the extent of experimental noise, which appears to be limited given the strong correlations observed (Figure 6a–c). Between conditions, the comparisons lead to weak correlations, showing important effects of the growth conditions on fitness strain ranking. For instance, there is no significant correlation when comparing proline and YPD media at 25°C (Spearman's ρ = −0.01883, *p*‐value = 0.8037, Figure 6d). These results reveal once again extensive genotype‐by‐environment interactions for fitness, to the extent that fitness values in one condition cannot be used to predict fitness values in certain other conditions.

## 4 DISCUSSION

The budding yeast *S. paradoxus* has been providing insights into the evolution and ecology of fungi over the past ten years. For example, studies have illustrated the role played by ecological and historical parameters in shaping the ecological distribution of North American populations (Eberlein et al., 2017; Leducq et al., 2016). Here, we developed a collection of barcoded strains to further empower these investigations. The availability of two barcoded copies of *S. paradoxus* strains allows the use of biological replicate for further experimentation and eventually the use of the different resistance markers for genetic studies. As the genome of a large fraction of these strains is fully sequenced (Leducq et al., 2016) or is being sequenced, the collection will provide valuable resources to address phenotypic and genomic questions. In addition, the collection offers the possibility to create haploids from the existing diploids, allowing to investigate aspects related to speciation and hybridization (Eberlein et al., 2017; Leducqet al., 2017, 2016). It is also possible to extend the collection by adding samples, as the barcode source is the *S. cerevisiae* KO collection that contains more than 4,000 unique barcodes, and many more could be created by designing new ones.

We showed that the insertion of the barcode does not significantly affect the growth of the strains in our tested conditions on solid medium (Figure 2). However, we observed that some strains show difference in fitness between the two cassette modules when grown in competition. These differences could arise from unwanted effects that occurred during transformation, including secondary mutations, multiple insertions, or genome instability that lead to the accumulation of variation among otherwise isogenic strains. Further investigation will be needed to assess the cause of this variation. A small fraction of barcode mis‐assignment could also contribute to these differences. Nevertheless, the majority show consistent behavior and thus provide an invaluable resource.

We revealed that even with a modest number of reads, significant fitness differences could be detected among strains and lineages. Our results are consistent with previous ones on the North American lineages *SpB*, *SpC*, and SpC*. It has been shown that the southern *SpB* and the intermediate SpC* lineages show increased fitness at high temperature (30–35°C) over the northern lineage *SpC* (Leducq et al., 2016). Our results are consistent with these observations. Furthermore, our analyses uncovered extensive genotype‐by‐environment interactions for fitness in these populations. Although the number of conditions tested is too small to allow for drawing general trends, these results suggest that no single lineage or strain would be able to outcompete the others in conditions that vary in space and/or time because the fitness ranking appears to be to a large extent independent among conditions, at least between the rich and defined conditions. These strains could be specialized in conditions that vary locally and across their respective geographical range, which would contribute to maintain a large diversity of genotypes in North America.

Finally, the *S. paradoxus* barcoded collection allows for the design of competition experiments to explore the characteristics of divergent populations within a species. Further investigations could use this resource to evaluate the impact of mixed culture on the growth rate and how the interaction among strains within the same lineage or among different lineages could impact the individual fitness. Furthermore, both biotic and abiotic factors shape the evolution of populations and communities. Experimental studies have shown the influence of competition on how specific microbial isolates cope with a novel environment or with a fluctuating environment (Bleuven & Landry, 2016; Lawrence et al., 2012; Osmond & de Mazancourt, 2013; Pekkonen, Ketola, & Laakso, 2013; Van Den Elzen, Kleynhans, & Otto, 2017). In its environment, *S. paradoxus* is surrounded by an abundant diversity of microbial species and its growth success will depend on the composition of these communities (Kowallik, Miller, & Greig, 2015). By using the *S. paradoxus *barcoded collection as a tractable and genetically well‐characterized model system, it will become possible to study the selective pressures of abiotic as well as biotic factors that shape the species evolution in highly controlled conditions. Finally, further research could benefit from coupling experiment in controlled conditions with tools such as our *S. paradoxus *barcoded collection and methods that are developed to measure microbial fitness and persistence in nature (Anderson et al., 2018; Boynton, Stelkens, Kowallik, & Greig, 2017).

## CONFLICT OF INTEREST

The authors have no conflict of interest.

## AUTHORS CONTRIBUTION

AKD, IGA, and CRL designed the experiment; CB, AKD, IGA, and GN acquired the data; CB, GN, and HM analyzed and interpreted the data; CB wrote the manuscript with input from CRL and GN. All authors contributed and agreed on the content of the final version.

## ETHICS STATEMENT

None required.

## Data Availability

Raw sequencing data are available at Bioproject number PRJNA453404 and SRA Accession: SRP150766.
