# Supplementary material for: A collection of barcoded natural isolates of Saccharomyces paradoxus to study microbial evolutionary ecology
Source: Microbiologyopen. 2018 Dec 19;8(7):e00773. doi: 10.1002/mbo3.773 (PMC6612553; doi:10.1002/mbo3.773)
Supplement: Supplementary file 1 [file MBO3-8-e00773-s001.docx]

**Supplemental Information for:**

**A collection of barcoded natural isolates of *Saccharomyces paradoxus* to study microbial evolutionary ecology.**

Clara Bleuven, Alexandre K. Dubé, Guillaume N’Guyen, Isabelle Gagnon-Arsenault, Hélène Martin, Christian R. Landry

**Table of Contents:**

| **Table S1** | Page 1 (Excel sheet) |
| --- | --- |
| **Table S2** | Page 2 |
| **Table S3** | Page 4 |
| **Table S4** | Page 5 |
| **Table S5** | Page 6 |
| **Table S6** | Page 7 |
| **Table S7** | Page 8 |
| **Figure S1** | Page 9 |

**Table S1 |** List of barcoded strains: Name of the strain, lineage, sample location, nature of the substrate, reference, cassette module associated with the barcode, barcode obtained by Sanger sequencing, comment on the result (identification of the potential mutated barcodes and non-legible barcodes likely due to sequencing error), *S. cerevisiae* strain from the deletion collection used as a source of barcode, in case of failure to amplify the first strain we also report the *S. cerevisiae* strain used as replacement, the barcode associated to the *S. cerevisiae* strain as sequenced by Filteau *et al*. (2017) and their official version from the Deletion collection (Giaever *et al.* 2002) (when the two are different, the barcode in bold correspond to the sequenced barcode of the *S. paradoxus* strain), whether the full genome has been sequenced and presence in the pools analysed in this study. (see Sup file TableS1_Supplemental_Information.x

**Table S2** | List of oligonucleotides used in this study.

| **Name** | **Sequence** | **F/R** | **Lineage specificity** | **Protocol** | **Information** |
| --- | --- | --- | --- | --- | --- |
| CLO5-22 | CATCCTTATAGGCAGCAATCAATTCCATCTAAACTTCAACGATGTCCACGAGGTCTCT | F | *SpB - SpD* | PCR UPTAG barcode amplification | Homology to *S. paradoxus* lineage at the HO locus |
| CLO5-23 | CATCCTTATAGGCAGCAATCAATTCCATCTAAACTTTAACGATGTCCACGAGGTCTCT | F | *SpC - SpC** | PCR UPTAG barcode amplification | Homology to *S. paradoxus* lineage at the HO locus |
| CLO5-24 | GTTCGGATGTGATGTGAGAAC | R | All | PCR UPTAG barcode amplification | Homology to resistance cassette module |
| CLO5-25 | CGTACGCTGCAGGTCGAC | F | All | PCR Amplification of resistance cassette | Homology to barcode module/resistance cassette module |
| CLO5-26 | AATTAATTACATAACAATTTTTTTTTATAATATACATATTATCGATGAATTCGAGCTCG | R | *SpB - SpD* | PCR Amplification of resistance cassette | Homology to *S. paradoxus* lineage at the HO locus |
| CLO5-27 | CATTTATTACATACAACTTTTTTTTAATAATATACATATTATCGATGAATTCGAGCTCG | R | *SpC - SpC** | PCR Amplification of resistance cassette | Homology to *S. paradoxus* lineage at the HO locus |
| CLOP95-F3 | GTGGCTTTTGCTTCTATTACGACCATTACCTCTAAACTCACATCCTTATAGGCAGCAATC | F | *SpB - SpD* | PCR fusion | Barcode amplification to fuse with resistance cassette and put at HO locus |
| CLOP95-F4 | CTATAAGATCATTTTTATCCAAAATATTAAAATTTACATTAATTAATTACATAACAATTT | R | *SpA - SpB - SpD* | PCR fusion | Barcode amplification to fuse with resistance cassette and put at HO locus |
| O5-23 | CATCCTTATAGGCAGCAATCAATTCCATCTAAACTTTAACGATGTCCACGAGGTCTCT | F | *SpC -SpC** | PCR fusion | Barcode amplification to fuse with resistance cassette and put at HO locus |
| O5-27 | CATTTATTACATACAACTTTTTTTTAATAATATACATATTATCGATGAATTCGAGCTCG | R | *SpC - SpC** | PCR fusion | Barcode amplification to fuse with resistance cassette and put at HO locus |
| CLOP95-F5 | GTGGCTTTTACTTCTATTACAACCATTACCTCTAAATCCACATCCTTATAGGCAGCAATC | F | *SpC - SpC** | PCR fusion | Replaced by O5-23 |
| CLOP95-F6 | AGTCTTATAAGATCATTTTAATCCAAAATATTAAAATTTACATTTATTACATACAACTTT | R | *SpC - SpC** | PCR fusion | Replaced by O5-27 |
| CLOP48-C11 | ACAGAAGCTTGTTGAAGCGC | F | All | colony PCR | Confirmation of the HO locus |
| CLO5-28 | CAATGGTTTAGTAGGAACCC | R | All | colony PCR | Confirmation of the HO locus |
| O1-37 | CGGTAAGCCGTGTCGTCAAG | R | All | colony PCR | Diagnostic of NAT integration in the genome |
| O1-38 | GTCGCGGTGAGTTCAGGCTT | R | All | colony PCR | Diagnostic of HPH integration in the genome |

**Table S3 |** Summary of barcoding efficiency during the rounds of transformation. The number of transformants corresponds to strains that have successfully inserted the barcode module before validation by Sanger sequencing.

| **Barcode** | **Lineage** | **Round** | **Transformants** | **Initial number of parental strains** | **% of successful transformation** |
| --- | --- | --- | --- | --- | --- |
| NAT | *SpB* | 1 | **147** | 247 | *59.5* |
|  |  | 2 | **56** | 63 | *88.9* |
|  | *SpC* | 1 | **42** | 64 | *65.6* |
|  |  | 2 | **12** | 22 | *54.5* |
|  |  | 3 | **5** | 9 | *55.6* |
|  | *SpC** | 1 | **17** | 50 | *34.0* |
|  |  | 2 | **18** | 33 | *54.5* |
|  |  | 3 | **1** | 6 | *16.7* |
|  | *SpD* | 1 | **3** | 9 | *33.3* |
|  |  |  |  |  |  |
| HPH | *SpB* | 1 | **112** | 247 | *45.3* |
|  |  | 2 | **72** | 77 | *93.5* |
|  | *SpC* | 1 | **40** | 64 | *62.5* |
|  |  | 2 | **17** | 23 | *73.9* |
|  |  | 3 | **4** | 6 | *66.7* |
|  | *SpC** | 1 | **36** | 50 | *72.0* |
|  |  | 2 | **5** | 14 | *35.7* |
|  |  | 3 | **3** | 5 | *60.0* |
|  | *SpD* | 1 | **5** | 9 | *55.6* |

**Table S4** | PCR cycles of the barcoding protocol

| Barcode amplification | 5 min at 95°C; 30 cycles of 20s at 90°C; 15s at 64°C; 30s at 72°C followed by a final step of 5min at 72°C. | | | | | | | |
| --- | --- | --- | --- | --- | --- | --- | --- | --- |
|  |  |  |  |  |  |  |  |  |
| Amplification cassette | 5min at 95°C; 30 cycles of 20s at 98°C; 15s at 64°C; 1min at 72°C followed by a final step of 5min at 72°C. | | | | | | | |
|  |  |  |  |  |  |  |  |  |
| PCR fusion | 5min at 95°C; 30 cycles of 20s at 98°C; 15s at 64°C; 1min at 72°C followed by a final step of 5min at 72°C. | | | | | | | |
|  |  |  |  |  |  |  |  |  |
| Barcode insertion | 5min at 95°C; 35 cycles of 30s at 95°C; 30s at 55°C; 1min at 72°C followed by a final step of 5min at 72°C | | | | | | | |
|  |  |  |  |  |  |  |  |  |
| Barcode sequencing | 3 min at 95°C; 5 cycles of 20s at 98°C; 15s at 52°C; 15s at 72°C followed by 20 cycles of 20 s at 98°C and 15s at 75°C. | | | | | | | |
|  |  |  |  |  |  |  |  |  |

**Table S5** | Comparison of colony size (log_2_) between the HPH, NAT modules barcoded strains and their parental strains on solid YPD media. The Kruskal-Wallis tests show no significant differences in the two tested temperatures. Eight replicates were tested for each parental and barcoded strain with *SpB* = 151 strains, *SpC* = 52 strains, *SpC**=32 strains and *SpD* = 2 strains.

| **Temperature (°C)** | **Lineage** | **df** | **Chi-squared** | **p-value** |
| --- | --- | --- | --- | --- |
| 25 | *SpB* | 2 | 1.6521 | 0.4378 |
| 35 |  | 2 | 2.8563 | 0.2398 |
| 25 | *SpC* | 2 | 0.59138 | 0.744 |
| 35 |  | 2 | 0.84501 | 0.6554 |
| 25 | *SpC** | 2 | 0.57375 | 0.7506 |
| 35 |  | 2 | 0.2635 | 0.8766 |
| 25 | *SpD* | 2 | 0.125 | 0.9394 |
| 35 |  | 2 | 3 | 0.2231 |

**Table S6** | Number of strains used in the analysis of the competitive assay. The numbers correspond to the strains kept after sequence filtering and sorting in the different conditions. Strains with the two Tag1-HPH and Tag2-NAT barcode modules and with more than 100 reads at t_1_ in each condition were considered.

| **Lineages** | **YPD at 25°C** | **YPD at 35°C** | **Proline at 25°C** |
| --- | --- | --- | --- |
|  | Detected strains number | Detected strains number | Detected strains number |
| *SpB* | 127 | 127 | 128 |
| *SpC* | 45 | 45 | 44 |
| *SpC** | 27 | 27 | 27 |
| *SpD* | 2 | 2 | 2 |

**Table S7** | Pairwise comparisons between the fitness of the *S. paradoxus* lineages in each condition. Lineage with no significant difference in fitness (>0.01) are in bold. The mean fitness of four replicates was tested for each lineage in each condition by Kruskal-wallis tests followed by Dunn post–hoc tests. Values with *SpD* were not tested because because only data for two strains were available.

| **Condition** | **Pairwise Comparisions** | **Chi-squared** | **p-value** |
| --- | --- | --- | --- |
| Proline 25 °C | | 72.618 | < 2.2e^-16^ |
|  | ***SpB - SpC*** |  | **0.025** |
|  | *SpB - SpC** |  | < 2e^-16^ |
|  | *SpC - SpC** |  | 2.4e^-08^ |
| YPD 25 °C | | 105.8 | < 2.2e^-16^ |
|  | *SpB - SpC* |  | 1.8e^-05^ |
|  | *SpB - SpC** |  | < 2e^-16^ |
|  | *SpC - SpC** |  | 1.3e^-07^ |
| YPD 35 °C | | 194.22 | < 2.2e^-16^ |
|  | *SpB - SpC* |  | < 2e^-16^ |
|  | *SpB - SpC** |  | 1.7e^-14^ |
|  | *SpC - SpC** |  | 0.00047 |
|  |  |  |  |

**
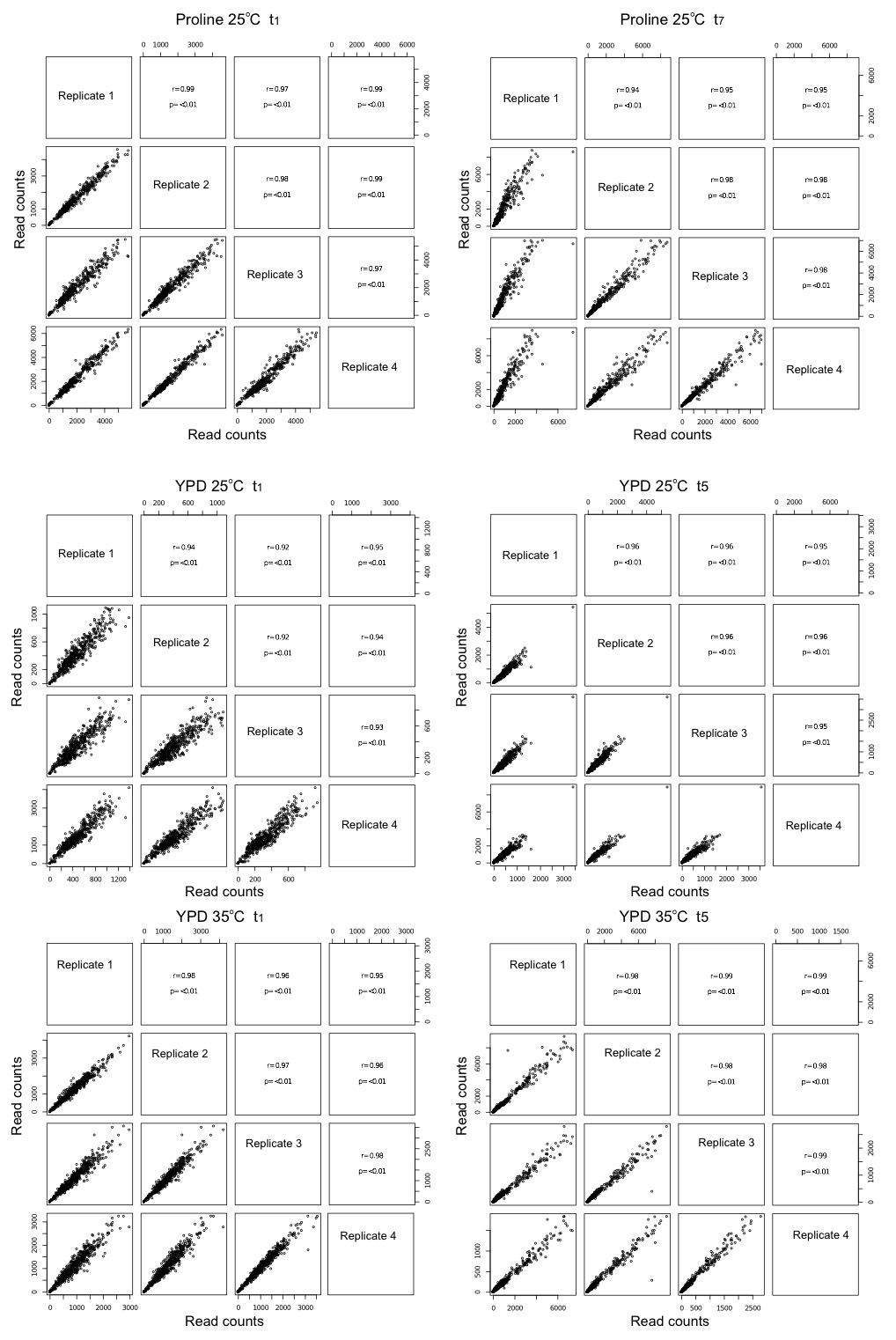
**

**Figure S1**. Correlation of read counts among replicates (Pearson’s r = 0.94-0.99, p-value <0.01).

**Supplementary references**

Filteau M, Charron G, Landry CR (2017) Identification of the fitness determinants of budding yeast on a natural substrate. *Isme j* **11**, 959-971.

Giaever G, Chu AM, Ni L*, et al.* (2002) Functional profiling of the Saccharomyces cerevisiae genome. *Nature* **418**, 387-391.
